# Splenic abscess due to fungal infection after kidney transplantation; a case report

**DOI:** 10.15171/jrip.2016.35

**Published:** 2016-07-12

**Authors:** Tahereh Malakoutian, Maliheh Yarmohamadi, Ronak Mohammadi, Mojgan Asgari, Reyhaneh Mahmoodian

**Affiliations:** ^1^Department of Nephrology, Hashemi Nejad Nephrology & Urology Center Hospital, Iran University of Medical Sciences, Tehran, Iran; ^2^Department of Internal Medicine, Kosar Hospital, Semnan university of Medical Sciences, Semnan, Iran; ^3^Department of Nephrology, Hashemi Nejad Nephrology & Urology Center Hospital, Iran University of Medical Sciences, Tehran, Iran

**Keywords:** Renal transplantation, Immunosuppressive therapy, Candidiasis, Splenectomy

## Abstract

Splenic abscess is one of the rare and potentially life-threatening complications after kidney transplantation. Splenic abscess generally occurs in patients who have immunodeficiency state. It becomes more important with the increased use of immunosuppressed drugs and organ transplantation. The clinical presentation of splenic abscess is insidious, often with constitutional symptoms. Left upper quadrant tenderness is an uncommon sign. Therefore, its diagnosis is difficult and requires a high degree of clinical suspicion. We report a case under renal transplantation with recurrent fungal infection in different organs with two episodes of fungemia who died after splenectomy.

**Implication for health policy/practice/research/medical education:**

Splenic abscess generally occurs in patients who have immunodeficiency state. It becomes more important with the increased use of immunosuppressed drugs and organ transplantation. We report a case of renal transplantation with recurrent fungal infection in different organs with two episodes of fungemia who died after splenectomy.

## Introduction

Splenic abscess is an uncommon disease with Incidence rate of 0.1%-0.7% in autopsy series (1). It has no specific clinical picture due to high mortality rate of lesion, it needs early detection and treatment. Although the most common causes of splenic abscess are bacterial pathogens but fungal splenic abscess has been reported in organ transplanted patients receiving immunosuppressive drugs with an increasing incidence rate from 0.8% to 25.8%. The most frequent pathogens are *Candida albicans, Aspergillus* and *Cryptococcus*. The diagnosis has become much more common in recent years as the population at risk has increased in size and more sensitive imaging techniques have been applied (2). A histologic diagnosis is often necessary while blood and tissue cultures may be falsely negative, particularly with candida infections (2). The treatment is usually splenectomy. However, anti-fungal therapy with close radiologic follow-up may be sufficient in some cases (3). Without prompt treatment, the infection is often fatal.

## Case Report

A-59-year-old woman presented to the hospital with fever, generalized weakness, and decreased appetite started from one week ago.

She was known case of end-stage renal disease due to autosomal dominant polycystic kidney disease that has underwent renal transplantation from a living unrelated donor six months ago in another center. Her medications including prednisolone, cyclosporine, and mycophenolate mofetil (MMF).

She had a history of raised serum creatinine and Candida pyelonephritis in her allograft kidney, one month after transplantation that had been confirmed by kidney biopsy and was treated with caspofungin and her immunosuppressive drugs was discontinued. At that time, she was discharged from hospital with good general condition and normal kidney function while she was receiving voriconazole and low dose corticosteroid. After two months, MMF 250 mg two times per day was added to her drugs. Her serum creatinine was between 1.1 mg/dL to 1.4 mg/dL. She came again with weakness and fever.

On physical examination, she was febrile with a pulse rate of 100 beat/min and blood pressure of 110/70 mm Hg. General physical examination did not reveal any abnormality. Her laboratory data revealed increased leukocyte count with majority of neutrophils, blood urea nitrogen; 92 mg/dL, serum creatinine; 7 mg/dL, hemoglobin level; 9.6 g/dL. Abdominopelvic sonography showed 50×48×35 mm^3^ heterogeneous splenic mass.

Antifungal therapy was begun and patient underwent splenectomy on third day of her admission.

**Figure 1:**
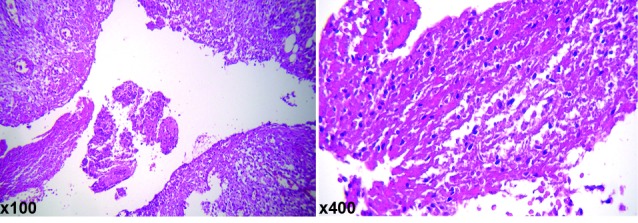
Sections of spleen show large amount of fungal yeast and hyphae (x200).

Spleen biopsy showed abscess formation, including numerous fungal element suggestive for candida infection (Figure 1). Unfortunately, she died after splenectomy because of fungal septicemia.

## Discussion

Pyogenic splenic abscess particularly fungal type is a rare condition with a tendency to occur in organ transplanted patients who are immunosuppressed. As the symptoms of systemic fungal infections are nonspecific, diagnosis requires a high index of suspicion.

Different organs may be affected by fungal infections. Among them splenic involvement in the form of abscess or micro-abscesses has the least prevalence. In an autopsy study of 39 patients with disseminated fungal infection, spleen involvement with low prevalence rate 19% was found (4).

Some articles pointed to an autopsy incidence of 0.1% to 0.7% for splenic abscess (1). Until now, 600 cases of abscess of the spleen are reported in world’s literature (5).

First case of candida splenic abscess in a renal transplant recipient was reported by Nemec et al that was treated successfully by splenectomy and amphotericin B despite a lengthy illness, the patient recovered with preservation of renal function (6).

Considering the rarity of this entity and high rate of mortality that reached by 47% based on some reports (7), we reported this case to share our experience in diagnosis and treatment of the diseases with other health centers to find a way for successful management of the patients. On the other hand, as the frequency of invasive fungal infections are increasing due to improving the survival of immunocompromised patients, the diagnostic and treatment approaches require more attention.

Typical clinical manifestations are fever and left upper quadrant pain with or without splenomegaly. However, some patients do not have these classic features (8). Splenic abscess may be accompanied by a left-sided pleural effusion (9) or by splenic infarction if it was due to septic emboli (10). There is no specific laboratory test for the diagnosis. Blood cultures usually are negative and histopathologic demonstration of Candida organisms in tissue specimens is necessary for a definitive diagnosis. Our patient was an immunosuppressed patient who had been experienced one episode of fungal pyelonephritis one month after kidney transplantation and was recovered with preservation of renal function at that time. Despite discontinuation and then minimization of her immunosuppressive drugs, fungal splenic abscess was occurred and she died just one day after splenectomy.

Splenectomy has long been considered as the standard treatment of bacterial and fungal splenic abscess (10). Recent reports suggest that patients with fungal abscesses can be treated medically using antifungal agents and needle aspiration without splenectomy (11).

There are some encouraging reports of treatment with amphotericin B therapy alone (12) or fluconazole in the case of resistance to amphotericin B (13), as an efficacious and less toxic alternative to amphotericin B (14). Also there are some reports of treatment with administration of caspofungin (15) and corticosteroid therapy as an adjuvant to antifungal treatment (16).

There are also some reports of successful treatment by inserting a catheter into the portal vein under ultrasonic-guidance and administration of amphotericin B through it (17,18).

Surgical options include percutaneous aspiration, percutaneous catheter drainage, open drainage and splenectomy (1,19).

Percutaneous computed tomography-guided drainage should be limited to patients with unilocular abscess cavities with a discrete wall and thin fluid collections without septations. As fungal abscesses usually are multiple and the patients have usually disseminated fungal infections due to their immunodeficiency state, they do not obtain the criteria for CT-guided drainage.

Splenic abscess is usually managed by a combination of antibiotic therapy and splenectomy (2,20). Although CT-guided percutaneous aspiration is occasionally successful, but it seems that surgery still remains the standard treatment (2).

According to the rarity of this disease, randomized clinical trials to assess the treatment modalities has been impossible, report of this case and same ones help the clinicians to emerge the data in the management of their patients by reviewing the literature.

## Conclusion

This case presents an unusual and abnormal location of involvement of opportunistic infection in a renal transplant patient. Early diagnosis and intervention (medical and surgical) can reduce unpleasant complications and decrease mortality.

## Authors’ contribution

All authors contributed equally to the manuscript.

## Conflicts of interest

The authors declared no competing interests.

## Ethical considerations

Ethical issues (including plagiarism, data fabrication, double publication) have been completely observed by the authors.

## Funding/Support

The authors have no financial relationships relevant to this article to disclose.
